# Lateral Flow Immunochromatography Assay for Detection of Furosemide in Slimming Health Foods

**DOI:** 10.3390/foods10092041

**Published:** 2021-08-30

**Authors:** Yingying Li, Haihuan Xie, Jin Wang, Xiangmei Li, Zhili Xiao, Zhenlin Xu, Hongtao Lei, Xing Shen

**Affiliations:** Guangdong Provincial Key Laboratory of Food Quality and Safety, South China Agricultural University, Guangzhou 510642, China; wzlyy@stu.scau.edu.cn (Y.L.); xiehaihuan@stu.scau.edu.cn (H.X.); wangjin940810@stu.scau.edu.cn (J.W.); lixiangmei12@scau.edu.cn (X.L.); scauxzl@scau.edu.cn (Z.X.); jallent@163.com (Z.X.); hongtao@scau.edu.cn (H.L.)

**Keywords:** furosemide, Au nanoparticles, lateral flow immunochromatography, slimming health food

## Abstract

In recent years, furosemide has been found to be abused in slimming health foods. There is an urgent need for a simpler, faster method for detecting furosemide in slimming health foods. In this study, a rapid, convenient and sensitive lateral flow immunochromatography (LFIA) based on Au nanoparticles (AuNPs) was established for the first time. Under optimal conditions, the qualitative limit of detection (LOD) of the AuNPs-based LFIA was 1.0~1.2 μg/g in slimming health foods with different substrates. AuNPs-LFIA could specifically detect furosemide within 12 min (including sample pretreatment) and be read by the naked eye. The developed AuNPs-LFIA showed high consistency with liquid chromatography with tandem mass spectrometry (LC-MS/MS), and no false positive or false negative results were found in spiked slimming health foods, proving that the AuNPs-LFIA should be accurate and reliable. The AuNPs-LFIA reported here provides a serviceable analytical tool for the on-site detection and rapid initial screening of furosemide for the first time.

## 1. Introduction

Overweight and obesity are the main risk factors for many chronic diseases. According to a World Health Organization (WHO) report, 39% of the global population aged 18 and over were overweight in 2016, while 13% (male 11%, female 15%) were obese [1]. In order to lose weight quickly, people are increasingly turning to the consumption of slimming dietary supplements. To pursue economic benefits, some slimming dietary supplements have been illegally adulterated with synthetic drugs to obtain obvious short-term effects [2,3].

According to the current legislation of the European Union (EU), the USA and China, synthetic drugs are not allowed in dietary supplements due to their harmful side effects [4]. However, some unscrupulous traders continue to illegally add drugs to slimming dietary supplements to increase the weight loss effect for the purpose of promoting sales, especially diuretics, appetite suppressants, gastrointestinal lipase inhibitors, energy expenditure agents and laxatives. Diuretics are common adulterants in slimming health foods. They accelerate the excretion of water from the body, causing the illusion of weight loss. Consumers could purchase and take slimming health foods containing diuretics without knowing it. Overdosing these products can produce side effects such as fluid and electrolyte abnormalities as well as acid–base disturbances, which may cause severe arrhythmia and increase the risk of death from arrhythmia [5]. Furosemide is one of the most effective diuretic medications available. It acts directly on the kidneys to increase urine output and the urinary excretion of sodium [6]. Oral formulations of furosemide are commonly used to treat edema, congestive heart failure, renal failure and hypertension [7]. In recent years, it has often been found to be illegally added to weight loss health foods. In 2020, the Institute for Drug Control of Suzhou, China, tested 84 batches of slimming health foods, and the illegal addition of furosemide was detected in 13 batches of samples (positivity rate, 15.5%) [8]. Therefore, it is necessary to establish a detection method for the illegally added drug furosemide in health foods.

At present, various methods for detecting furosemide in slimming products have been reported, including capillary electrophoresis (CE) [9,10,11], ion migration spectrometry (IMS) [12], ion-pair chromatography (IPC) [13,14], liquid chromatography-tandem mass spectrometry (LC-MS/MS) [15], high performance liquid chromatography (HPLC) [16] and ultra-high-pressure liquid chromatography (UHPLC) [17,18]. All these methods rely on expensive equipment, which is difficult to operate and requires trained operators. Although their instrumental methods are accurate, they can not meet the requirements of rapid on-site inspection. The rapid detection of furosemide mainly includes electrochemical and immunoassay methods. Electrochemical analysis methods are mainly used to detect furosemide in urine and drugs, and have a good detection speed, detection sensitivity and detection throughput [6,19,20,21]; however, all of them lack simplicity and selectivity to the negatively charged furosemide. Immunoassay is a rapid analysis method that is currently widely used. By now, only two enzyme-linked immune sorbent assays (ELISAs) have been reported for detecting furosemide in horse plasma and milk [22,23,24]. However, ELISA also involves complex testing procedures and long incubation times, so it remains a laboratory-based platform unsuited to on-site detection. A simpler and faster on-site detection method is needed for monitoring the growing number of slimming products.

Lateral flow immunochromatography assay (LFIA), which is simple, rapid and low-cost, has been widely used in food safety, environmental monitoring and medical diagnosis in recent years [25,26,27,28,29]. AuNPs have many advantages as a mature labeling material, such as simple preparation, short labeling time, good stability and low cost [30,31]. Thus, they are favored by manufacturers and occupy more than 90% of the label market in LFIA [32]. In this paper, a convenient AuNPs-LFIA detecting furosemide with good sensitivity and specificity was developed for the first time and proved to be efficient for application in the detection of furosemide in slimming health foods.

## 2. Materials and Methods

### 2.1. Materials

Furosemide, goat anti-rabbit IgG (secondary antibody), bovine albumin (BSA), ovalbumin (OVA), *N*,*N*-dimethylformamide (DMF), 1-(3-fdimethylaminopropyl)-3-ethylcarbodiimide hydrochloride (EDC) and ProClin 300 were purchased from Sigma-Aldrich (St. Louis, MO, USA). Chloroauric acid, trisodium citrate and polyvinyl pyrrolidone (PVP) were purchased from Sinopharm Chemical Reagent Co., Ltd. (Shanghai, China). Anti-furosemide antibodies and coating antigens were prepared in our own laboratory. Other chemicals were purchased from Guangzhou Chemical Reagent Co., Ltd. (Guangzhou, China). All reagents were of analytical grade or higher purity. The nitrocellulose filter (NC) membrane (CN95) was obtained from Sartorius Stedim Biotech GmbH (Goettingen, Germany). The sample pad (blood filtration membranes) and the polyvinyl chloride (PVC) backing plate (SMA31-40) were purchased from Shanghai Liangxin Co., Ltd. (Shanghai, China).

### 2.2. Instruments

An FEI/Talos L120C transmission electron microscope (TEM) (Thermo Scientific, Waltham, USA) was used to observe the morphologies of nanoparticles. The zeta potential was measured by a Zetasizer Nano ZS90 (Malvern Panalytical, UK). The XYZ 3060 Dispensing Platform (BioDot, Irvine, CA, USA) was used to spray antigen and secondary antibodies onto the NC membrane. The strip cutter ZQ 2000 (Kinbio Tech, Shanghai, China) was used to cut test strips into suitable sizes. LC-MS/MS was carried out on an AB QTRAP4500 triple quadrupole mass spectrometer (SCIEX, Framingham, MA, USA).

### 2.3. Preparation of Coating Antigen

The coating antigen was obtained by furosemide coupled with cationized ovalbumin (cOVA). cOVA is obtained by modifying OVA with ethylenediamine. Furosemide contains a carboxyl group, which could be directly coupled with cOVA by the active ester method to produce a coating antigen. Furosemide (1 equiv.), N-Hydroxy succinimide (NHS) (1.5 equiv.) and 1-(3-fdimethylaminopropyl)-3-ethylcarbodiimide hydrochloride (EDC) (1.5 equiv.) were dissolved in 200 μL of *N*,*N*-dimethylformamide (DMF). The mixture was stirred at 4 °C for 6 h, and then centrifuged at 2500× *g* for 10 min. The supernatant was added dropwise to cOVA (20 mg) in 5 mL of PBS (phosphate-buffered saline, 0.01 M, pH 7.4). The conjugate mixture was stirred at 4 °C overnight and dialyzed against PBS (0.01 M, pH 7.4) for 3 days at 4 °C to remove the uncoupled free hapten and non-reacted reactants. The obtained conjugate was used as coating antigen.

### 2.4. Preparation of AuNPs

The AuNPs were produced by reducing HAuCl_4_ with sodium citrate according to a previous method, which was modified as described below [33]. An amount of 8 mL of 1% chloroauric acid solution was quickly added into 200 mL of boiling ultrapure water under continuous stirring. When the solution boiled again, 9.25 mL of 1% trisodium citrate was added. The solution was then stirred and heated for another 10 min. After cooling, transmission electron microscopy and UV–visible absorption spectrometry were used to characterize the morphologies of AuNPs. The prepared AuNPs were stored at 4 °C for use.

### 2.5. Preparation of AuNPs–Abs Conjugated Probe

**Figure 1:**
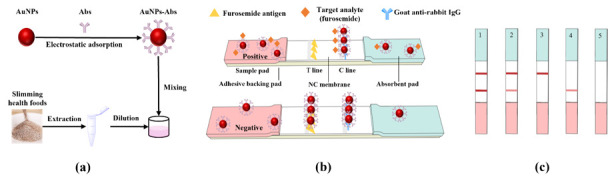
Schematic of the Au nanoparticles lateral flow immunochromatography (AuNPs-LFIA) for detecting furosemide in slimming coffee. (**a**) Preparation of the signal probe AuNPs–Abs and the sample treatment solution. (**b**) The structure and test procedure of the AuNPs-LFIA test strip. C line: control line (goat anti-rabbit immunoglobulin G, IgG) and T line: test line (furosemide coating antigen). (**c**) Schematic diagram of AuNPs-LFIA strip test results: 1, negative result; 2, weak positive result; 3, positive result; 4–5, invalid result.

The AuNPs–Abs conjugated probe was prepared via electrostatic adsorption between AuNPs and antibodies (Figure 1a). The optimal labeling pH and the antibody amount were adjusted by checkerboard titration. A suitable amount of 0.2 M K_2_CO_3_ was added into the AuNPs solution to adjust the pH value. Anti-furosemide antibody dissolved in 100 μL of 0.01 M PB (phosphate buffer solution, pH 7.4) was quickly added into the pH-adjusted AuNPs solution. The mixture was reacted for 10 min at room temperature. Then, 20 μL of 20% BSA was added and incubated for 20 min to block excess binding sites on the AuNPs. After centrifuging at 10,000× *g* and 4 °C for 10 min, the supernatant was discarded, the bottom red precipitate was resuspended with 200 μL of resuspension buffer (0.005 M borate buffer solution, pH 8.0, containing 0.5% BSA, 5% trehalose for protecting antibody, 0.5% Tween-20 both for a better release AuNPs–Abs probe and to adjust the chromatography speed, 0.3% PVP as a steric stabilizer or capping agent to protect the AuNPs–Abs against agglomeration, and 0.03% ProClin 300 to prevent metamorphism), and finally stored at 4 °C for further use.

To better reflect the performance of the AuNPs–Abs conjugated probe, a series of influencing parameters were optimized, including the pH value, the concentration and dilution buffer of antibody and antigen, and the resuspension buffer of AuNPs–Abs. The optimal conditions were selected according to the T line color intensity and sensitivity (inhibition rate, (1-OD_positive_/OD_negative_) × 100%).

### 2.6. Strip Assembly

The test strip of the LFIA was composed of an NC membrane, a sample pad, an absorbent pad and an adhesive backing pad (Figure 1b). The sample pad was saturated with 0.05 M PB (pH 7.4, containing 0.5% BSA, 0.5% Tween-20, 0.3% PVP and 0.03% ProClin 300) and dried for 12 h at 37 °C. The coating antigen and goat anti-rabbit IgG, which served as the test line and the control line (T line and C line), were diluted with 0.05 M CB (carbonate buffer solution, pH 9.6) and 0.02 M PB (pH 7.4), respectively, to an appropriate concentration, and then sprayed on the NC film with a volume of 0.8 μL/cm. The T line was 8 mm from the bottom of the NC film, and the distance between the T line and the C line was 6 mm. Then the prepared NC membrane was dried at 37 °C for 12 h. Finally, all parts were pasted on a PVC baking card, cut into 3.5 mm-wide strips and placed in a sealed bag with desiccant.

### 2.7. Sample Preparation

Four slimming health foods with different substrates (capsule, coffee, tea and tablet) were obtained from the local market, and were previously confirmed to be free of furosemide using LC-MS/MS. The outer shell of the capsule was removed to obtain the powder. The coffee, tea and tablets were taken out of their packing bags and ground into powder. An amount of 1.00 g of sample was added into a 10 mL centrifuge tube containing 4 mL of methanol and mixed on a vortex mixer for 2 min. Then the mixture was centrifuged at 4000× *g* for 3 min. To obtain the sample solution, 200 μL of the supernatant was added to 800 μL of 0.2 M PB (pH 7.4).

### 2.8. Test Procedure

In this study, the vertical operation mode was used in the strip testing process. We added 150 μL of sample solution and 5 μL of AuNPs-labeled conjugated probe to the microwell, and the probe was gently pipetted back and forth to evenly disperse it in the sample solution. After incubating for 3 min at room temperature, the test strip was inserted immediately and vertically into the microwell. After reacting for another 4 min, the test strip was removed from the microwell. The qualitative result was simply read with the naked eye. The signal intensity of the T line and C line was read and obtained by ImageJ software. In more detail, the optical density of the test zones (negatives and positives) in grayscale mode was measured by the ImageJ software to obtain the color intensity.

### 2.9. Sensitivity

The cut-off value was utilized to determine the sensitivity of the developed LFIA test strips by assessing the concentration of the furosemide in a series of spiked samples with test strips. The cut-off value of the assay is defined as the furosemide level that causes the T line to disappear completely. The sensitivity in actual samples with different substrates was evaluated separately. Sample preparation was carried out according to the previous description. Blank slimming health food samples were spiked with furosemide standard solution (100 µg/mL, diluted in methanol) to the final concentrations of 0 (control), 0.1, 0.2, 0.4, 0.6, 0.8, 1.0 and 1.2 µg/g. Each level was tested three times (*n* = 3). 

### 2.10. Specificity

To evaluate the specificity of the proposed method, furosemide analogues which may be illegally added to slimming health foods, including hydrochlorothiazide, metolazone, bumetanide, acetazolamide, torasemide and ethacrynic acid, were added to the sample solution (1.2 μg/g) for detection.

### 2.11. Method Confirmation

Four different substrates of slimming health foods were selected to validate the accuracy of the AuNPs-LFIA. The spiked concentrations were chosen according to the sensitivity of samples of different substrates. The spiked scalar was the furosemide concentration corresponding to 0.25, 0.5, 1 and 2 times the cut-off value. A total of 16 spiked samples were tested. Each sample was combined with 4 mL methanol, mixed on a vortex mixer for 2 min, and then centrifuged at 4000× *g* for 3 min. Half of the supernatant was diluted 5 times with PB and tested with test strips, and the other half was diluted 5 times with methanol and passed through a 0.22 μm filter membrane, then tested with LC-MS/MS. Each sample was tested three times (*n* = 3).

Chromatographic separation of LC-MS/MS was performed on a Waters CORTECS T3 column (2.1 × 100 mm, 2.7 μm). The column temperature was 30 °C. Mobile phase A was an aqueous solution containing 0.1% formic acid, and phase B was an acetonitrile solution containing 0.1% formic acid, which was used for gradient elution. The sample volume was 1 µL.

### 2.12. Analysis of Blind Samples

We purchased 16 blind samples of slimming health foods from a local Guangzhou market and analyzed them by the established AuNPs-LFIA and LC-MS/MS. The samples were pretreated following the method above. The established AuNPs-LFIA was used to detect furosemide in the blind samples, and the results were confirmed by LC-MS/MS.

## 3. Results and Discussion

### 3.1. Characterization of AuNPs and AuNPs–Abs

**Figure 2:**
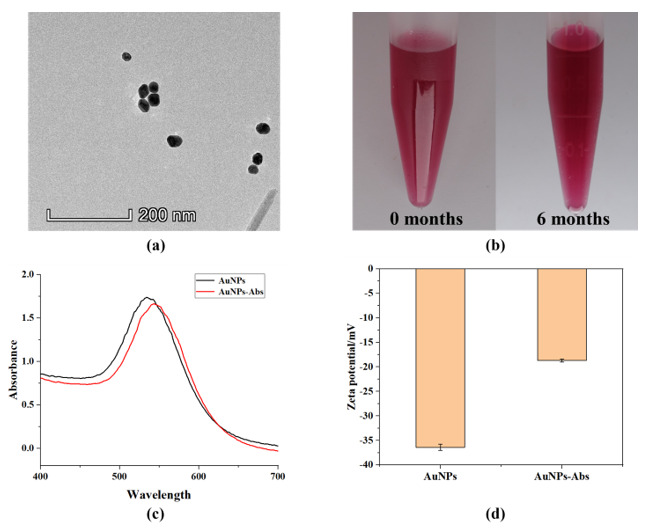
Characterization of Au nanoparticles (AuNPs) and Au nanoparticles-antibodies (AuNPs–Abs). (**a**) Transmission electron microscopy images of AuNPs; (**b**) the results of naked-eye observation of AuNPs at different times; (**c**) the UV–visible absorption spectra of AuNPs and AuNPs–Abs; (**d**) the zeta potential of AuNPs and AuNPs–Abs.

It can be seen from the obtained TEM images (Figure 2a) that the prepared AuNPs were monodisperse spherical particles. The diameter of the AuNPs was about 35 nm, which was consistent with the required size. In the stability test of AuNPs, the AuNPs solution stored at 4 °C for 6 months did not appear to aggregate or precipitate (Figure 2b), indicating that its dispersion stability could last for at least six months. According to the results of UV–visible absorption spectrometry (Figure 2c), the AuNPs exhibited maximum absorbance at a wavelength of 534 nm. After combination with antibodies, the maximum absorbance shifted to 543 nm, indicating the formation of the AuNPs–Abs conjugate. The combination of AuNPs and antibodies is generally believed to be the result of electrostatic attraction [34]. Under certain conditions, AuNPs have a negative surface charge, and the antibodies have a positive charge on the surface [35]. From the performance of the zeta potential, when the negatively charged AuNPs were combined with the positively charged antibodies, the result showed a decrease in the AuNPs zeta potential. Figure 2d shows that the zeta potential decreased from 36.4 mV to 18.7 mV after conjugation, which further confirms that the antibodies coupled with AuNPs successfully.

### 3.2. Optimization

#### 3.2.1. pH Value

**Figure 3:**
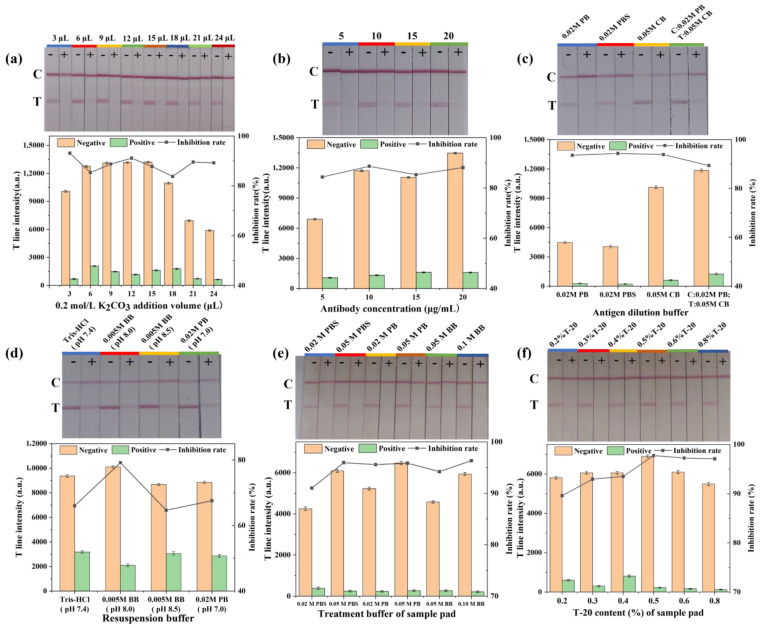
Optimization results of Au nanoparticles lateral flow immunochromatography (AuNPs-LFIA). The LFIA test strip results correspond to the T line intensity obtained by ImageJ software. (**a**) Effect of the pH value represented by 0.2 M K_2_CO_3_ volume in 1 mL AuNPs; (**b**) antibody concentration of AuNPs–Abs conjugated probe; (**c**) effect of the dilution buffer of coating antigen and second antibody; (**d**) effect of the resuspension buffer of AuNPs–Abs probe; (**e**) effect of ion type and ion concentration in the sample pad treatment solution; (**f**) effect of Tween-20 content in the sample pad treatment solution. −, Negative; +, positive.

The pH values during the reaction were critical to the efficacy of AuNPs–Abs conjugates. Theoretically, the pH of the reaction should be slightly higher than the isoelectric point of the protein. Below the isoelectric point, the antibodies may flocculate and cause the aggregation and precipitation of AuNPs–Abs, which would decrease the accuracy and cause false negatives. Above the isoelectric point, the adsorption effect is limited due to the charge repulsion between the antibodies and the AuNPs, and the color of the test strip would turn light. According to signal intensity and sensitivity, the optimal pH of AuNPs solutions was 7.8, corresponding to 12 µL of 0.2 M K_2_CO_3_ solution added (Figure 3a).

#### 3.2.2. Antibody Concentration

In general, the signal intensity is proportional to the concentration of antibodies, but excess antibodies would affect the sensitivity of the LFIA. In order to screen the optimal concentration of antibodies, antibodies of different concentrations (5, 10, 15 and 20 µg/mL) were added to AuNPs solution to synthesize AuNPs–Abs conjugates. The results show that the highest sensitivity of strip assay was obtained at an antibody concentration of 10 µg/mL (Figure 3b).

#### 3.2.3. Dilution Buffer 

The dilution buffer of antigen and goat anti-rabbit second antibody had a great influence on color intensity as a result of the effects on the absorption of protein in the NC membrane caused by the ion type and pH value. In this study, 0.02 M PB (pH 7.4), 0.02 M PBS (phosphate-buffered saline, pH 7.4) and 0.05 M CB (pH 9.6) were selected as the dilution buffers. The results (Figure 3c) show that when the antigen was diluted in 0.05 M CB and the second antibody was diluted in 0.02 M PB, a stronger signal could be obtained.

#### 3.2.4. Resuspension Buffer

The resuspension buffer affected the stability of AuNPs–Abs. In this study, Tris-HCl (pH 7.4), 0.005 M borate buffer solution (pH 8.0), 0.005 M borate buffer solution (pH 8.5) and 0.02 M PB (pH 7.0) were chosen as the resuspension buffers. We found that the resuspension buffers of Tris-HCl and PB could lead to AuNPs–Abs coagulation on the second day, which may have been caused by inappropriate pH values for the dissolution of the AuNPs–Abs probe. In the end, 0.005 M borate buffer solution (pH 8.0), which had the highest assay sensitivity, was chosen as the resuspension buffer (Figure 3d).

#### 3.2.5. Sample Pad Treatment Solution

The sample pad plays a crucial role in reducing the interference of the sample matrix and affected the binding of the labeled probe on the NC membrane, thereby affecting the color intensity and sensitivity of the test strip. Here we mainly evaluated the different buffer and ion concentrations and the Tween-20 content of the sample pad pretreatment solution. As shown in Figure 3e, the higher ion concentration of the sample pad treatment solutions led to better color intensity. In general, Tween-20 improved the fluidity of the sample pad. However, when the flow rate was too fast, it was not conducive to the T-line capture of the AuNPs–Abs. When the flow rate was too slow, it increased the detection time. It can be seen from Figure 3f that as the content of Tween-20 increased, the color intensity of the LFIA first showed an increasing trend, and then decreased. By comparison, we chose a sample pad treatment solution formulation with 0.05 M PB and 0.5% Tween-20 content.

### 3.3. Sensitivity 

**Figure 4:**
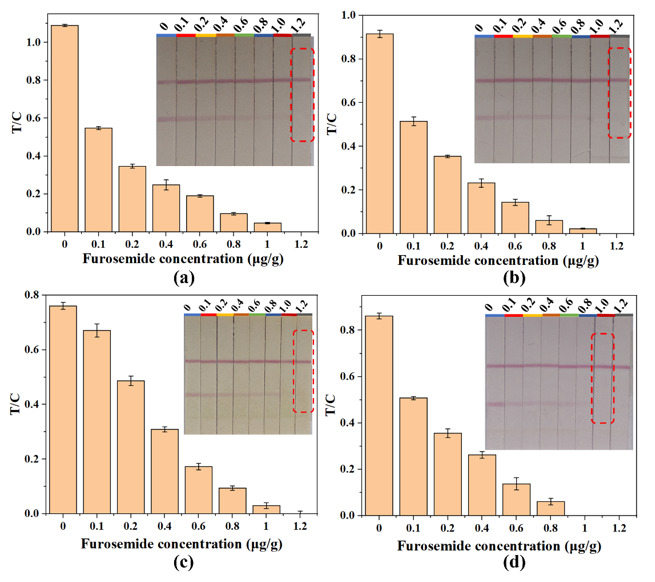
Sensitivity evaluation of the Au nanoparticles lateral flow immunochromatography (AuNPs-LFIA) for furosemide in slimming health foods of different substrates. Red rectangular boxes indicate the color intensity at the cut-off concentrations. Results for determination of furosemide in the slimming capsule samples (**a**), slimming coffee samples (**b**), slimming tea samples (**c**) and slimming tablet samples (**d**).

The qualitative performance of the AuNPs-LFIA was evaluated by the cut-off value. A series of furosemide with different concentrations were spiked into the negative slimming health food samples. Figure 4 shows that the color intensity of T line became weaker with increasing furosemide concentration. When the concentration of furosemide was 0 ng/g, a vigorous color intensity could be seen with the naked eye on the T line, and the color intensity became weaker when a higher concentration of furosemide was added. According to the testing result, the cut-off value was 1.2 μg/g in capsule, coffee and tea samples, and it was 1.0 μg/g in tablet samples. However, the effective dosage of furosemide for an adult is 20–40 mg/day [36]. Therefore, slimming health foods would need to add at least 20 mg of furosemide to the daily dosage to achieve significant weight loss, which is far greater than the LOD of the AuNPs-LFIA. Additionally, compared to the LOD of the HPLC-MS/MS method (2.7 μg/g) established by the Chinese government (BJS 201710) for illegally added furosemide detection in health foods, the established AuNPs-LFIA showed higher sensitivity and achieved on-site detection using a simple operation procedure.

### 3.4. Specificity 

**Figure 5:**
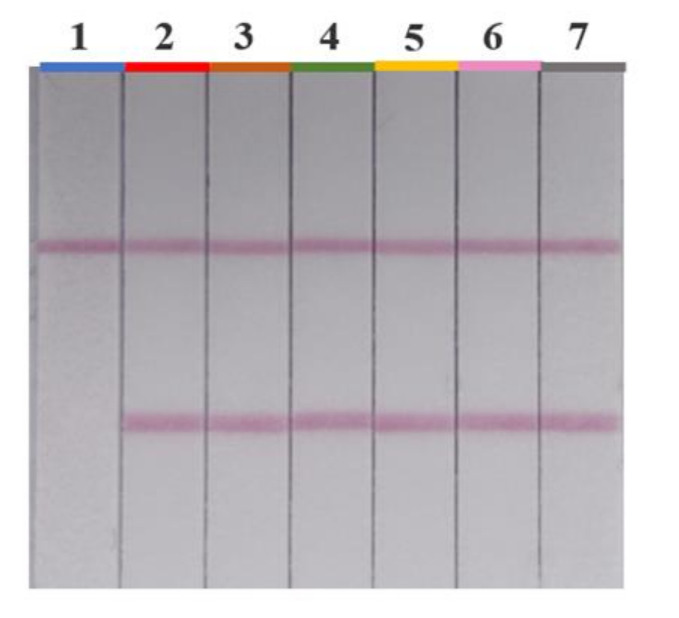
Specificity evaluation results of the Au nanoparticles lateral flow immunochromatography (AuNPs-LFIA): 1, furosemide; 2, hydrochlorothiazide; 3, metolazone; 4, bumetanide; 5, acetazolamide; 6, torasemide; 7, ethacrynic acid.

The developed AuNPs-LFIA was used to detect six furosemide analogues, including hydrochlorothiazide, metolazone, bumetanide, acetazolamide, torasemide and ethacrynic acid at a 1.2 μg/g level. The results showed that the test strip did not detect the other drugs at all, indicating that the LFIA had a high specificity for furosemide detection in slimming health foods (Figure 5). 

### 3.5. Confirmation by LC-MS/MS

LC-MS/MS was employed for the method confirmation. The detection results of AuNPs-LFIA were consistent with LC-MS/MS in all 16 spiked samples. No false-positive or false-negative results were found. The recoveries for furosemide in spiked samples were from 75.83% to 104.53%, with the CVs ranging from 0.09% to 4.92% (Table 1), indicating that the established LFIA is reliable and could be used for large-scale sample screening on-site.

### 3.6. Analysis of Blind Samples

The 16 slimming health foods purchased in a Guangzhou market were detected by AuNPs-LFIA, and the results showed that all were furosemide-negative samples (Table 2). The results were confirmed and consistent with LC-MS/MS. Although no positive sample was found in this survey due to the sample size and strict supervision in Guangzhou, the results still indicate that the established AuNPs-LFIA is accurate and suitable for the detection of different substrate samples.

## 4. Conclusions

This study developed a sensitive AuNPs-LFIA for the rapid detection of the illegal adulterant drug furosemide that is sometimes found in slimming health foods for the first time. By optimizing a series of parameters that may affect the performance of the AuNPs-LFIA, the sensitivity for furosemide detection was higher than the detection limit of the HPLC-MS/MS method formulated by the Chinese government for the detection of illegal furosemide addition to health food. The sample preparation and test operation of the developed AuNPs-LFIA is 12 min in total, and the procedure is simpler and faster than other existing methods for furosemide detection. In conclusion, the developed AuNPs-LFIA could be applied as an on-site rapid detection method for the screening of furosemide in slimming health foods.

## Figures and Tables

**Table 1:** Comparison of the detection results of the Au nanoparticles lateral flow immunochromatography (AuNPs-LFIA) and the liquid chromatography with tandem mass spectrometry (LC-MS/MS) in slimming health foods spiked with furosemide (*n* = 3).

Sample	Spike Level (μg/g)	LC-MS/MS (μg/mL)	Recovery (%)	CV (%)	AuNPs-LFIA Result
coffee	0.30	0.346 ± 0.006	115.39	1.75	−−−
	0.60	0.707 ± 0.009	117.81	1.23	±±±
	1.20	1.379 ± 0.026	114.95	1.89	+++
	2.40	2.781 ± 0.014	115.90	0.49	+++
capsule	0.30	0.302 ± 0.005	100.80	1.56	−−−
	0.60	0.649 ± 0.014	108.10	2.22	±±±
	1.20	1.196 ± 0.059	99.64	4.92	+++
	2.40	2.295 ± 0.013	95.63	0.55	+++
tea	0.30	0.247 ± 0.004	82.25	1.70	−−−
	0.60	0.512 ± 0.016	85.35	3.05	±±±
	1.20	1.048 ± 0.032	87.37	3.02	+++
	2.40	2.127 ± 0.026	88.61	1.22	+++
tablet	0.25	0.200 ± 0.004	80.18	2.09	−−−
	0.50	0.379 ± 0.011	75.83	2.86	±±±
	1.00	0.879 ± 0.006	87.90	0.65	+++
	2.00	1.862 ± 0.002	93.11	0.09	+++

−, Negative; +, positive; ±, weakly positive.

**Table 2:** Comparison of the detection results of the Au nanoparticles lateral flow immunochromatography (AuNPs-LFIA) and the liquid chromatography with tandem mass spectrometry (LC-MS/MS) in slimming health foods purchased in a Guangzhou market (*n* = 3).

Blind Sample	LC-MS/MS	AuNPs-LFIA	Blind Sample	LC-MS/MS	AuNPs-LFIA
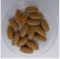 Sample 1	ND	---	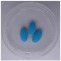 Sample 9	ND	−−−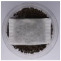 Sample 2	ND	---	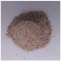 Sample 10	ND	−−−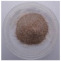 Sample 3	ND	---	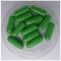 Sample 11	ND	−−−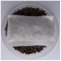 Sample 4	ND	---	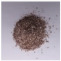 Sample 12	ND	−−−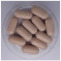 Sample 5	ND	---	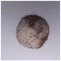 Sample 13	ND	−−−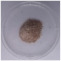 Sample 6	ND	---	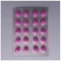 Sample 14	ND	−−−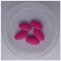 Sample 7	ND	---	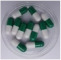 Sample 15	ND	−−−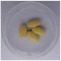 Sample 8	ND	---	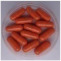 Sample 16	ND	−−−

ND, Not detected; −, negative.
